# The effects of Chinese Buddhist meditation tradition: the impact of nature observation and literary creation

**DOI:** 10.3389/fpsyg.2025.1615963

**Published:** 2025-08-01

**Authors:** Tiankai Liang, Minkai Sun, Seiko Goto

**Affiliations:** ^1^Faculty of Environmental Science, Nagasaki University, Nagasaki, Japan; ^2^School of Architecture and Urban Planning, Suzhou University of Science and Technology, Suzhou, China

**Keywords:** Chinese Buddhist temple, relaxation, nature, literature, mental state, meditation

## Abstract

**Introduction:**

After integrating with indigenous Chinese culture, Chinese Buddhist meditation traditions expanded beyond classical rock meditation to include new practices. This study examines the physiological and psychological effects of nature observation and literary creation within Chinese Buddhist meditation.

**Methods:**

Experiment 1 recruited 30 participants and used observation duration, heart rate, the Profile of Mood States (POMS), and a supplemental questionnaire to compare relaxation effects across water (LS), forest (FS), and rock (RS) landscapes at a Buddhist temple. Experiment 2 recruited 30 new participants and introduced a poetry-creation task in the most relaxing landscape (LS) to test additional effects.

**Results:**

The water LS significantly prolonged observation duration [LS: 379.835 ± 47.528 vs. FS: 210.656 ± 15.284 vs. RS: 272.157 ± 25.450, 95% CI (65.638, 272.719), *p* = 0.000, ηp^2^ = 0.443, 1-β = 0.985] and induced greater heart rate reduction (72.4 vs. 78.1 bpm at baseline, *p* = 0.001). POMS scores showed LS most improved negative moods (e.g., Depression-Dejection: −1.47 ± 0.38 vs. FS +1.07 ± 0.37, *p* < 0.001).

**Conclusion:**

Chinese Buddhist practices integrating water landscapes and poetry composition optimize relaxation (heart rate reduction: −7.3% in LS) and cognitive engagement, offering evidence-based insights for mental health interventions.

## 1 Introduction

In recent years, mental health challenges have become a global concern, with the global prevalence of anxiety and depressive disorders increasing by 25% during the first year of the pandemic alone (Santomauro et al., 2021). With the ongoing exploration of traditional culture, various classical ancient meditation practices are gradually being integrated into modern lifestyles, offering diverse solutions to contemporary mental health challenges. Traditional meditation practices have garnered worldwide attention, and exploring solutions for contemporary mental health issues from a multicultural perspective is gradually gaining interest, though such attention has yet to form a systematic theoretical or practical framework (Kabat-Zinn, 2003). This gap highlights the necessity of continuously delving into traditional practices to meet modern mental health needs.

When Buddhism spread from India to China, Chinese Buddhist meditation practices mirrored those in India, involving wall-facing meditation (Lomas, 2017) or visualizing the Western Pure Land while reciting scriptures day and night (Stevenson, 2007), praying for the Buddha's help to attain enlightenment and Buddhahood (Ingram, 1973). However, influenced by Chinese indigenous philosophies, China developed Chan Buddhism (Ren, 1994), a unique sect that emphasizes achieving enlightenment through personal effort, without excessive reliance on the Buddha's help or scripture recitation (Davis, 2014). This method broke away from traditional practices, initiating innovations in meditation techniques and exhibiting broader inclusivity, allowing the philosophies of Confucianism and Taoism to be integrated into Chan Buddhist meditation practices (Zhu, 2019).

**Figure 1:**
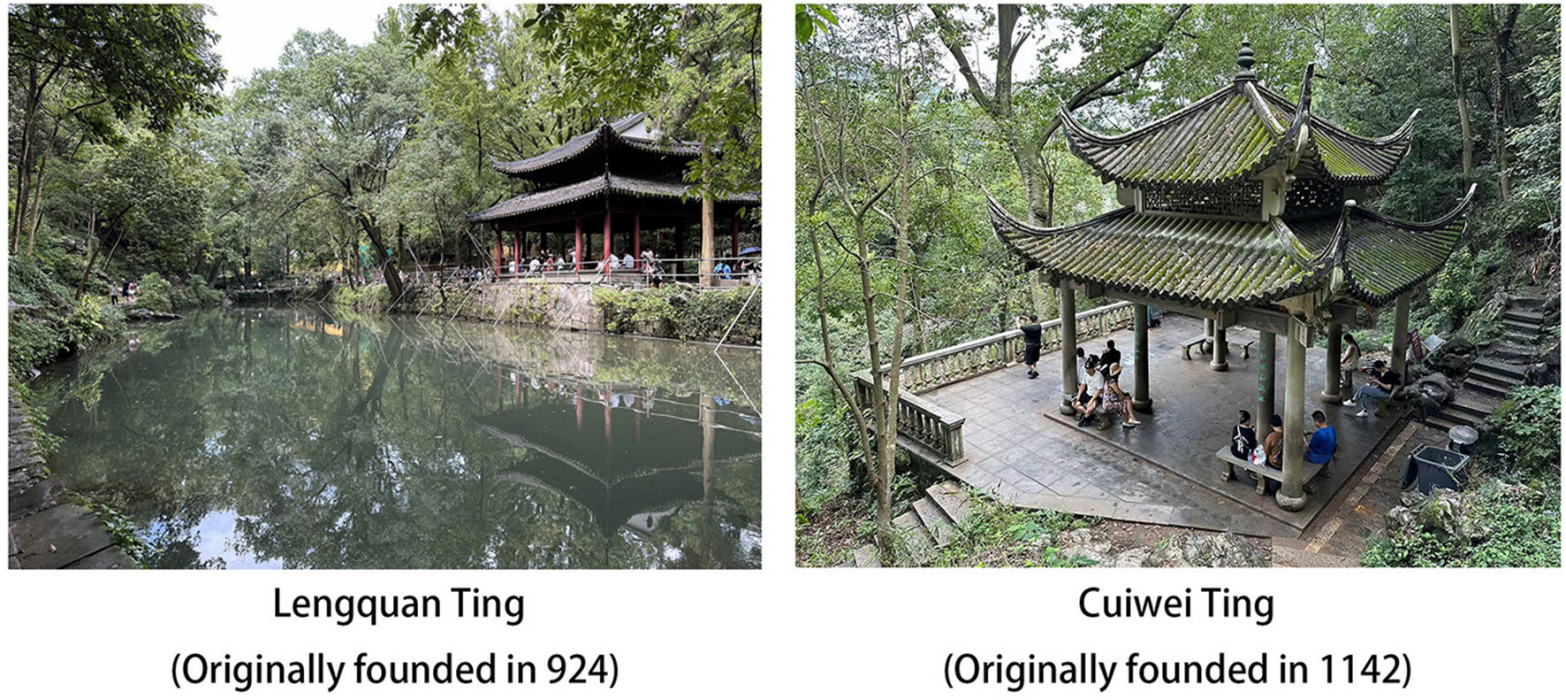
Meditative landscapes surrounding a Buddhist Temple in Hangzhou, China.

Influenced by Taoist meditation (Miller, 2003), the meditation method of Chan Buddhism integrated Taoist concepts like “the unity of human and nature” (天人合一) and “following the way of nature” (道法自然) into its attitude toward nature (Ren, 1994). The aim was to avoid artificial control while achieving harmony with nature through observation (Zhang, 2006). This meditation tradition involves selecting natural settings for appreciation as a focal point during meditation (Jian and Yi, 2024). In classical Chan Buddhist temples in China, viewing platforms (Ting 亭) were constructed in ancient times for the purpose of viewing natural landscapes (Figure 1), serving as preferred locations for rest, meditation, and leisure, thereby inducing relaxation effects (Yan and Zhu, 2023). Among natural environments conducive to meditation, rocks, water, and plants/forests are some of the most favored landscapes, as they are believed to foster an appreciation of nature's beauty, promote awakening and contemplation, and positively impact mental wellbeing (Guang, 2013; Karetzky, 1997; Parkes, 2008, 2021; Wang and Feng, 2023; Wang et al., 2020; Xu et al., 2023).

In addition to Taoist philosophy, which integrated Chinese Buddhist meditation with the natural environment, Confucian philosophy infused the literary spirit into Chinese Buddhist landscape meditation (Hsieh, 2010). With the influence of Confucianism, the creation of poems was integrated into the process of landscape appreciation during meditation, giving rise to Chan poet-monks (Protass, 2021). This process encourages individuals to describe the landscape using words while appreciating it, thereby deepening their understanding of the natural environment and simultaneously enhancing cognitive exercise (Chang, 2014). It also improves attention and cognitive agility, fostering a sense of leisure and vitality (Wu, 2016), which in turn reduces feelings of loss and fatigue, ultimately leading to a state of intellectual contemplation and relaxation (Yongwen, 2019).

In short, selecting specific locations in nature for viewing as well as composing poetry while observing the landscape are unique meditation practices in Chinese Buddhist temples, aimed at achieving a positive mental state and relaxation effects.

Chinese Buddhist meditation, shaped by the integration of Taoist and Confucian philosophies, offers a unique perspective on mental cultivation through nature observation and literary creation. Unlike classical Indian meditation, which emphasizes inward focus, Chinese practices encourage engagement with external stimuli—such as water, forests, and rocks—to achieve harmony between mind and environment (Zhang, 2006). These practices may provide a culturally resonant and scientifically viable alternative for stress reduction and cognitive enhancement. However, despite their historical significance, the physiological and psychological effects of these practices remain underexplored in empirical research. This lack of evidence limits their potential application in modern mental health interventions.

Therefore, this study conducted two experiments to investigate the following two research questions.

The first experiment: in a traditional site of Buddhist mediation in China, which element among water, rock, and forest landscapes has the most optimal relaxation effect on individuals?

The second experiment: since composing nature poetry is part of the Chinese Buddhist meditation tradition, how does composing poetry in the mind while viewing a specific landscape lead to a state of relaxation and what additional effects does it produce?

By answering these questions, this study aims to provide new perspectives on linking traditional Chinese practices with modern mental health needs, while delivering evidence-based foundations for culturally adapted interventions. The findings may also inform the design of therapeutic settings and meditation programs that integrate natural elements and creative expression.

## 2 Methods

The human research protocol in the study was approved by the Ethics Committee of Nagasaki University.

### 2.1 Experimental site

**Figure 2:**
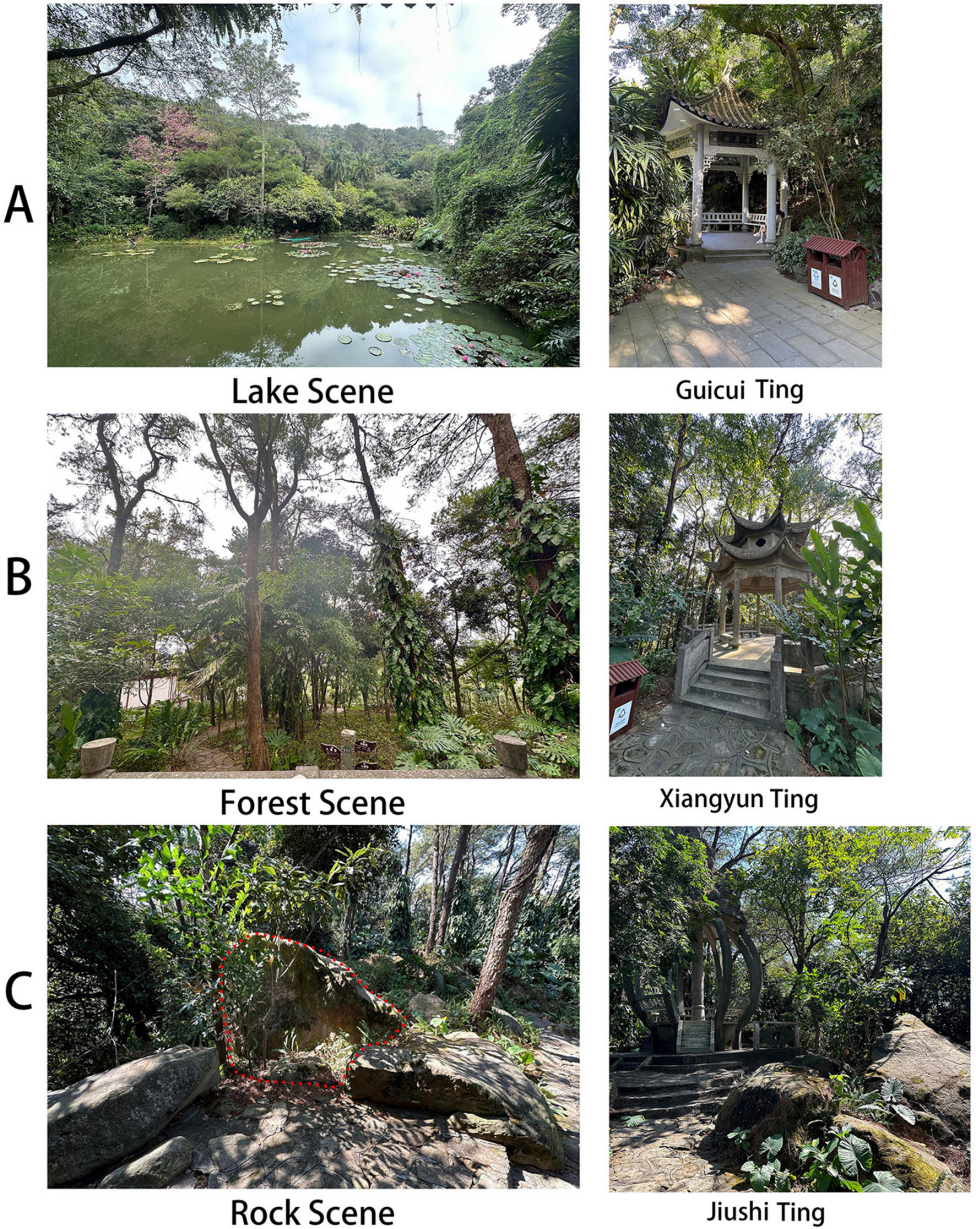
The three types of experimental sites selected for Experiment 1 (The area within the red dashed line in **(C)** indicates the region participants were instructed to focus on).

**Figure 3:**
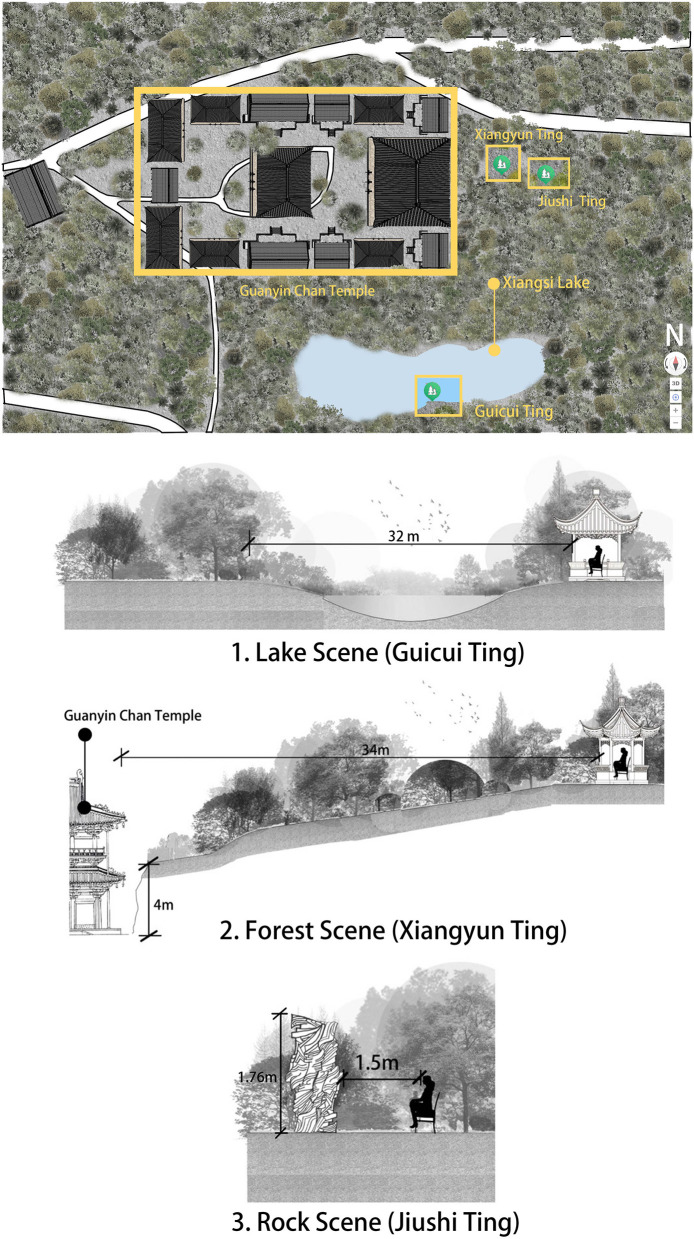
The locations of the three types of experimental sites.

For Experiment 1, three experiment sites were selected in Guanyin Chan Temple, located at the summit of Qingxiu Mountain in Nanning City, Guangxi, China (Figures 2, 3). Guanyin Chan Temple, the largest Chan Buddhist temple in Guangxi covering an area of approximately 15 acres, was built during the Song Dynasty (960-1127). It is surrounded by a rich natural environment, enveloped by the 13.54 square kilometers of forested and mountainous terrain of Qingxiu Mountain. According to information obtained from inquiries with relevant departments of the Nanning municipal government, three viewing platforms were built after 1949 on the request of monks of Guanyin Chan Temple to appreciate landscapes (rocks, water and forest), which became the sites for Experiment 1 (Figure 2).

The first platform is called Guicui Ting (Figure 2A), situated on the south of Xiangsi Lake (Figure 3). From Guicui Ting, one can observe a scene of Xiangsi Lake with a background of trees; such as *Small-leaved Fig, Bougainvillea, Silver Birch, Arenga Pinnata, Agarwood Tree, Screw Pine, Hardy Water Canna*, and *Morning Glory* (Figure 2A). This scene was referred to as the Lake Scene (LS).

The second platform is called Xiangyun Ting, located on the east of Guanyin Chan Temple and the west of Jiushi Ting (Figure 3). The Xiangyun Ting is located in the mountainside, surrounded by woods, and serves as a viewing point for observing the forest scenery. From Xiangyun Ting, one can observe various plants, such as *Fir Tree, Bamboo, Elephant Ear, Golden Pothos, Ash Tree, Osmanthus Tree, Camphor Tree*, and *Longan Tree*, with a view of Guanyin Chan Temple (Figure 2B). This scene was referred to as the Forest Scene (FS).

The third platform is called Jiushi Ting, which is a viewing platform for appreciating the rocks around the platform (Figures 2C, 3). Since the distance between Jiushi Ting and the rocks was too far to distinguish individual rocks, a chair was placed 1.5 meters away from the tallest rock (1.76 meters high and 2.6 meters wide, the rock within the red dashed lines in Figure 2C) to allow participants to recreate an environment similar to that of a Chan Buddhist monk meditating while facing a rock. The surface of the rock was weathered over time and covered with moss (Figure 3). This scene was referred to as the Rock Scene (RS).

For Experiment 2, the site with the best results from Experiment 1 was selected.

### 2.2 Participant recruitment

A priori power analyses were conducted using G^*^Power 3.1 for both experiments. For Experiment 1 (repeated measures ANOVA with 3 conditions: LS/FS/RS), we assumed a medium-large effect size (*f* = 0.35, based on pilot data), α = 0.05, power (1-β) = 0.80, correlation among repeated measures = 0.5, and non-sphericity correction ε = 1. The analysis indicated a required sample size of 24 participants, which we increased to 30 to account for attrition and enhance sensitivity. For Experiment 2 (paired-sample *t*-test comparing NP/WP conditions), we assumed a medium effect size (^*^d^*^ = 0.50, conservative estimate for poetry-task interventions), α = 0.05, power = 0.80, and correlation between pairs = 0.5, yielding a required sample size of 27 participants. We increased this to 30 participants per experiment to account for potential attrition and enhance sensitivity to smaller effects, while maintaining feasibility for controlled field experiments at heritage sites (Sun and Bao, 2025). This sample size exceeds the minimum recommendations for within-subjects psychophysiological research (Brysbaert, 2019) and aligns with comparable meditation-environment studies (Goto et al., 2020; Lymeus et al., 2017).

Experiment 1 recruited 30 participants (13 males and 17 females), including 20 undergraduate students and 10 employees from a construction company, with an average age of 33.6 ± 15.6 years. Experiment 2 recruited a group of 30 participants (15 males and 15 females), all of whom were employed professionals, including staff from construction companies, teachers, and employees from accounting firms, with an average age of 38.5 ± 10.8 years. The exclusion criteria were presence of heart problems, and < 20/400 vision (in U.S. units) corrected with eyeglasses or contact lenses. All participants were either studying or working in China.

### 2.3 Measurement methods

#### 2.3.1 Heart rate

An increase in heart rate indicates emotional tension or an increased risk of illness, while a decrease signifies emotional relaxation and improvement (Goto et al., 2020; Liang et al., 2024). Therefore, this study used heart rate as a measure to determine whether participants experienced physiological relaxation effects after viewing the scenes. A finger-mounted pulse plethysmograph monitoring device (Iworx IWX/404 and PT100 model) was employed to record heart rate values (bpm) during landscape observations. The device detects the pulse rate by monitoring blood volume changes in the finger and records the average heart rate every 10 s as a single observation. The signals were transmitted to a laptop positioned behind the participant, and heart rate data were recorded and analyzed using the software provided by the manufacturer.

The heart rate was measured over the same duration as the participants' landscape viewing experiment. The duration of the viewing experiment was not time-constrained and was determined by the participants themselves.

#### 2.3.2 Profile of mood states, 2nd edition–adult (POMS 2–A)

The POMS 2–A is a self-report assessment of mood that is used to capture transient and fluctuating feelings, or relatively enduring affect states in adults aged 18 years or older (Lin et al., 2014). The tool is a self-administered adjective rating scale composed of 35 items with a score ranging from 0 to 4; it measures seven mood states: Anger-Hostility (AH), Confusion-Bewilderment (CB), Fatigue-Inertia (FI), Depression-Dejection (DD), Tension-Anxiety (TA), Vigor-Activity (VA), and Friendliness (F). The participants completed the POMS 2-A before and after observing the three sites. The POMS manual was used to quantitatively calculate the T-scores and assess mood states. As the participants' native language was Chinese, the Chinese translation of the questions was used.

To ensure the reliability and validity of the Chinese version of POMS 2-A in our study, we conducted psychometric analyses on the current sample. Internal consistency was assessed using Cronbach's alpha coefficient for each of the seven mood subscales. Confirmatory factor analysis (CFA) was performed to examine the structural validity of the seven-factor model. Criterion validity was evaluated by examining the correlations between POMS subscale scores and heart rate changes, given that heart rate is an established physiological indicator of relaxation.

Previous research has shown that using POMS in landscape observation can effectively reflect participants' psychological and emotional states (Goto et al., 2017, 2020; Liang et al., 2024). Additionally, to maintain consistency and establish a connection with our prior work, we employed POMS as the psychological measurement metric in this study.

#### 2.3.3 Supplemental questionnaire

After the experiment, a Supplemental Questionnaire was administered to the participants in Experiment 1, which included rated questions and post-experimental comments. The rated questions consisted of the following eight items: (1) Do you like this scene? (2) Would you like to visit this place again? (3) Did you feel relaxed after viewing the scene? (4) Do the colors of the scene attract you? (5) Does the material of the scene attract you? (6) Do the animals and plants in this scene attract you? (7) Do the details of the scene attract you? (8) Does the overall scene attract you? The rating scale consisted of five levels: “NO” (1 point), “A Little No” (2 points), “Neither” (3 points), “A Little Yes” (4 points), and “YES” (5 points). The post-experimental comments were open-ended responses provided by the participants based on their personal experiences, with no word limit. In Experiment 2, the Supplemental Questionnaire only required the completion of post-experimental comments and did not include the rated questions.

#### 2.3.4 Poem composition

In Experiment 2, the participants were asked to compose five lines of poetry based on the scene in the landscape viewing process and write them down after viewing the landscape. Generally, many Chinese Chan Buddhist monks compose poetic lines consisting of five lines during landscape meditation (Harris, 2007; Whyde, 2017; Yuan and Xianfeng, 2022), with the first line also serving as the title, introducing the overall context of the landscape scene. The second to fourth lines describe the details of the scene and the interaction between the poet and the landscape, while the final line carries the poet's emotional judgments (Chou, 1982).

The participants were allowed to write the lyrics freely in colloquial language and not required to adhere to the tonal patterns of Chinese classical poetry, but the content and form had to be equivalent to the poems written by Chinese Chan monks. Therefore, the participants were asked to compose a poem using the form below as a reference:

(1^st^ line) Describe the overall characteristics of the scene.
(2^nd^ line) Describe the first feature (any observed landscape element, such as water bodies, rocks, plants, etc.).
(3^rd^ line) Describe the second feature (any observed landscape element, such as water bodies, rocks, plants, etc.).
(4^th^ line) Describe the third feature (any observed landscape element, such as water bodies, rocks, plants, etc.).
(5^th^ line) Describe how your mood is affected by either individual landscape elements or the overall scene.

To address the qualitative evaluation of the poems, a standardized rubric was developed based on common criteria used in evaluating nature-based reflective poetry in previous literature (Chang, 2014; Moffett, 1983). The rubric included four dimensions: (1) use of descriptive language (e.g., adjectives, metaphors), (2) positive emotional expression (connection to nature, positivity of mental state), (3) reflection depth (e.g., personal or philosophical insight), and (4) structural coherence. Each dimension was rated on a 5-point scale (1 = very weak, 5 = very strong).

Three independent raters (each holding a master's degree in Chinese Language and Literature) were trained using this rubric. They evaluated all poems independently while blinded to participant information and experimental conditions. Inter-rater reliability was assessed using intraclass correlation coefficients (ICC). When the ICC results do not achieve good reliability, the testing will continue until the result exceeds 0.7 (McGraw and Wong, 1996), thereby ensuring the reliability of the independent raters.

### 2.4 Procedure

#### 2.4.1 Experiment 1

To avoid the influence of order, 30 participants were divided into three groups, A, B, and C, with 10 participants in each group. Each group watched LS, FS, and RS in different sequences. The experimental sequence for Group A was FS-LS-RS, for Group B was LS-RS-FS, and for Group C was RS-FS-LS. The experimental sessions for Group A took place on November 19th (FS), 20th (LS), and 21st (RS), 2024, from 10:10 AM to 1:10 PM. During the 3-day experimental period for Group A, the average temperature at Guanyin Chan Temple was 19.9°C (±3.5°C), with an average humidity of 62% (±12%); all 3 days were sunny. The experimental sessions for Groups B and C were conducted on November 23rd, 24th, and 25th, 2024. For Group B, the daily experiment time was from 9:00 a.m. to 12:00 p.m., with LS observed on the first day, RS on the second day, and FS on the third day. For Group C, the daily experiment time was from 1:00 p.m. to 4:00 p.m., with RS observed on the first day, FS on the second day, and LS on the third day. During the 3-day experimental period for Group B and Group C, the average temperature at Guanyin Chan Temple was 19.4°C (±3.7°C), with an average humidity of 61% (±13%). The weather was sunny on the 23rd and 24th, but cloudy on the 25th.

The experiments were organized by two individuals: an experiment supervisor and an experiment assistant. The supervisor and assistant set up a waiting area located 10 meters away from the experimental site, which was equipped with chairs and tables for participants to fill out questionnaires or wait before entering the experiment. Each participant was informed of the specific location of the waiting area 3 days prior to the experiment and was required to arrive on time according to their individual schedule.

Upon arrival at the waiting area, the participant was not allowed to proceed directly to the experimental site. Instead, the participant was required to sign the participation consent form, complete the pre-POMS questionnaire, and rest for 2 min in the waiting area. Afterward, the experiment assistant placed an eye mask on the participant and guided the them to a rotating chair with a height of 50 cm at the experimental site (Figures 2, 3). In both LS and FS, the distance between the rotating chair and the railing was 60 cm. Once seated, the experiment supervisor attached a heart rate monitor to the participant and informed the participant that there was no time limit for the observation. The supervisor also instructed the participant to raise the hand without the heart rate monitor if they wished to end the experiment. After the participant confirmed that they understood of the instructions, the supervisor and assistant simultaneously started the heart rate monitor, removed the participant's eye mask, and started the stopwatch to synchronize the timing with the heart rate monitor. This marked the beginning of the observational phase of the experiment. When the participant felt that the observation was sufficient, they raised the hand without the heart rate monitor. Simultaneously, the experiment supervisor stopped the heart rate monitor and stopwatch, and then removed the heart rate monitor from the participant's finger. Then, the participant gradually rotated the chair to face away from the scene and was guided by the experiment assistant to the waiting area to complete the post-POMS and Supplemental Questionnaire. Once the participant finished the questionnaires, their experiment was concluded, and the next participant began a new round of the experiment. Each participant's observation time (i.e., the heart rate monitor recording time) was pre-set to 20 min, which did not include the time spent filling out questionnaires and other activities. While one participant was engaged in observation and heart rate monitor recording, the next participant, with the assistance of the experiment assistant, completed various questionnaires, ensuring efficient use of time. After completing the experiments conducted at the three sites, each participant received a compensation of 120 Chinese yuan.

#### 2.4.2 Experiment 2

In Experiment 2, the participants were required to visit the same site twice. The second visit was scheduled at least 1 week after completing the first visit. The participants were divided into two groups (Group A and Group B), with 15 individuals in each group. The visit in which the participants were not required to create a five-line poem was referred to as the no poetry-making section (NP), while the visit with the poetry-making task was referred to as the poetry-making section (WP). Group A was required to first participate in the WP experiment, followed by the NP experiment after a gap of more than 1 week. Group B, on the other hand, was required to first participate in the NP experiment, followed by the WP experiment. Group A participated in the WP experiment on December 10, 2024, from 9:00 a.m. to 4:30 p.m., while Group B participated in the NP experiment on December 15, 2024, from 9:00 a.m. to 4:30 p.m. During the 2-day experimental period, the average temperature at Guanyin Chan Temple was 14.4°C (±3.6°C), with an average humidity of 34% (± 6%). The weather on both days was cloudy. After a gap of more than 1 week, Group A participated in the NP experiment on December 28, 2024, from 9:00 AM to 4:30 PM, while Group B participated in the WP experiment on December 29, 2024, from 9:00 AM to 4:30 PM. During the two-day experimental period, the average temperature at Guanyin Chan Temple was 13.2°C (± 4.7°C), with an average humidity of 34% (± 5%). The weather was sunny on both days.

The procedure of Experiment 2 did not include the POMS task. Although the omission of the POMS measure in Experiment 2 limits direct comparison of mood states between WP and NP, this decision was made based on practical constraints and methodological integrity. Specifically, during the WP (with-poetry) condition, participants were instructed to mentally compose poetry during the landscape viewing process and immediately write it down afterward. Our pilot observations and participant feedback revealed that requiring participants to complete the POMS immediately after viewing, but before writing down their poetic thoughts, caused cognitive interference and disrupted the poetic process. Conversely, administering the POMS after poem-writing introduced additional cognitive tasks (e.g., lexical retrieval, evaluative reflection), which could significantly confound the mood assessment, thereby undermining the validity of POMS scores as a reflection of pure landscape exposure. Therefore, to maintain the ecological validity of the poetry-writing experience and avoid task contamination, we chose to omit POMS in Experiment 2 and instead relied on objective physiological measures (i.e., heart rate), behavioral engagement (i.e., observation duration), and qualitative analyses of poetic content to evaluate psychological effects. Future studies may explore alternative strategies such as delayed mood assessments or implicit mood measures to address this limitation while preserving the integrity of literary-creative tasks.

In the NS experiment, once participants arrived at the waiting area, all procedures from the initial completion of various forms and questionnaires to the end of the experiment, including all tasks performed by the experiment supervisor and experiment assistant, were consistent with the procedures of Experiment 1, except that the rated questions in the Supplemental Questionnaire were removed, and participants were only required to provide post-experimental comments. In the WP experiment, the experiment assistant then presented a printed instruction sheet containing the structure of five sentences and instructed participants to create five sentences during the experiment based on these structures and write them down after the experiment ended. The post-experimental comments section in the Supplemental Questionnaire was omitted in the WP experiment.

### 2.5 Statistical analyses

#### 2.5.1 Experiment 1

For the heart rate data analysis, the average heart rate during the initial 30 s, the middle 30 s, and final 30 s of the observation duration were extracted for each participant. This was done to compare the differences in the average heart rate at the beginning, middle, and end of the duration across 30 participants in each experimental site. For further analysis, heart rate values were standardized, starting at 1 at the beginning of the duration (the initial 30 s), and normalized to 1 for subsequent times (the middle of the duration and the end of the duration; Goto et al., 2017, 2020; Liang et al., 2024). For each experimental site, a repeated measures analysis of variance was conducted to compare the mean heart rate values, using Bonferroni *post-hoc* tests with one within-subjects factor time (the beginning of the duration vs. the middle of the duration vs. the end of the duration).

As observation durations differed among participants, the average heart rate per minute was computed for each scene to examine heart rate trends under varying durations. The results were presented as scatter plots. Notably, the sample size for each minute was inconsistent. For instance, in this experiment, all participants viewed each scene for at least 1 min; thus, the sample size for the first 60 s was 30 (average values from 30 participants). However, in the LS, only two participants had an observation duration exceeding 18 min (1,080 s), resulting in a sample size of 2 for the 18th min in LS.

The mean values of observation duration, POMS, and questionnaire scores were compared by repeated measure analysis of variance using Bonferroni *post-hoc* tests with one within-subject factor site (LS vs. FS vs. RS). For the T-scores of the POMS 2-A, the difference between participants' responses before and after viewing the sites was calculated, and the magnitude of this after-minus-before (or before-minus-after) difference was compared across the experimental sites. For AH, CB, FI, DD, and TA, which represent negative emotions, the after-minus-before method was used; for VA and F, which represent positive emotions, the before-minus-after method was applied. After subtraction, smaller values indicate a better improvement in emotional response.

To examine the correlation between participants‘ voluntary engagement in viewing the landscape and relaxation effects, Pearson Correlations were used to analyze the relationship between the self-determined observation duration and POMS (the value of after-minus-before or before-minus-after). The value of observation duration was the total number of seconds for each participant. Additionally, the sum of scores from the first rated question and questions 4 through 8 was calculated and correlated with POMS measures to determine the relationship between participants' landscape preferences and relaxation effects. Since each participant's observation duration varied, their heart rate processes and the end time of the experiment also differed. To investigate the correlation between observation duration and heart rate, we divided the time into fixed intervals (e.g., 1–2 min) and performed a correlation analysis to determine the correlation between the average heart rate per minute and corresponding time intervals across different scenes. To understand the strength of the correlation between observation duration and relaxation effects while controlling for the effects of group order (Group A, Group B, and Group C), partial correlations were performed. Descriptive statistics and Shapiro–Wilk normality tests showed that all data were normally distributed.

Given the multiple correlation analyses conducted, we applied the Benjamini-Hochberg false discovery rate (FDR) correction to control for Type I errors in all correlation analyses. For *post-hoc* comparisons following repeated-measures ANOVAs, we used Bonferroni correction.

Significance was set at *p* < 0.05^*^, *p* < 0.01^**^, and *p* ≤ 0.001^***^. The results are presented as means ± SEM. Partial Eta squared (*ηp*^2^) was used to calculate effect sizes.

#### 2.5.2 Experiment 2

For the experimental site of Experiment 2, the evaluation metrics included the observation duration, heart rate, and Supplemental Questionnaire from each scene in Experiment 1. For observation duration, the average value across all 30 participants was used. For heart rate, the average normalized heart rate at the end of 30 s for all 30 participants was used. For the Supplemental Questionnaire, the average values for the eight questions for 30 participants were summed, and these summed results were used. Smaller values for heart rate indicate more positive outcomes, so the value of heart rate was reverse-coded in the calculations. The final score was calculated using the following formula:


$$\begin{array}{l} {\text{Final score = observation duration }}^{*}\text{ 40}\%\text{ + heart rate}\\ \;*\;40\%\;+{\text{ Supplemental Questionnaire }}^{*}\;20\%. \end{array}$$

The site with the highest final score was selected as the location for Experiment 2. The weighting distribution of each indicator was based on a comprehensive consideration of objective physiological measures (heart rate) and behavioral engagement (observation duration). The research showed that these metrics were of equal importance in explaining motivational outcomes (Cacioppo et al., 1993; Lang, 1995; Russell, 2003), so this study assigned them the largest and uniform weighting. To avoid the potential negative impact of subjective opinions and auxiliary information (Kahneman, 2011), the Supplemental Questionnaire was assigned a relatively small proportion.

To validate the robustness of the weight distribution (40% heart rate, 40% duration, 20% questionnaire), we conducted a sensitivity analysis testing three alternative weight schemes:

Scheme 1: equal weights (33.3% each).
Scheme 2: dominant physiological (50% heart rate, 30% duration, 20% questionnaire).
Scheme 3: dominant behavioral (50% duration, 30% heart rate, 20% questionnaire).

For the physiological responses, paired-sample *t*-tests were used to compare the differences in observation duration and heart rate (the initial 30 s, the middle 30 s, and the final 30 s) between the WP and NP experiments as well as for within-subject single-factor comparative analyses. Independent samples *t*-tests were used for between-subject single-factor comparative analyses.

To analyze the content of five-line poems in WP, the poetic content of each of the 30 participants was imported into Voyant Tools software for extracting useful keywords, which were then categorized into five groups: Landscape Objects, Colors for Describing Landscape Objects, Adjectives for Describing Landscape Objects, Psychological Reactions, and Nature Phenomena. To compare the differences in the number of keywords among participants in each category, the log (x + 1) function was applied to normalize the data and mitigate the impact of excessive zero values.

The method of correlation analysis was consistent with that in Experiment 1 (controlling variable: Group A and Group B). Descriptive statistics and Shapiro–Wilk normality tests showed that all data were normally distributed. Significance was set at *p* < 0.05^*^, *p* < 0.01^**^, and *p* ≤ 0.001^***^. Results are presented as means ± SEM. Cohen's d (*d*) was used to calculate effect sizes.

## 3 Experiment 1

### 3.1 Results

#### 3.1.1 Observation duration

**Figure 4:**
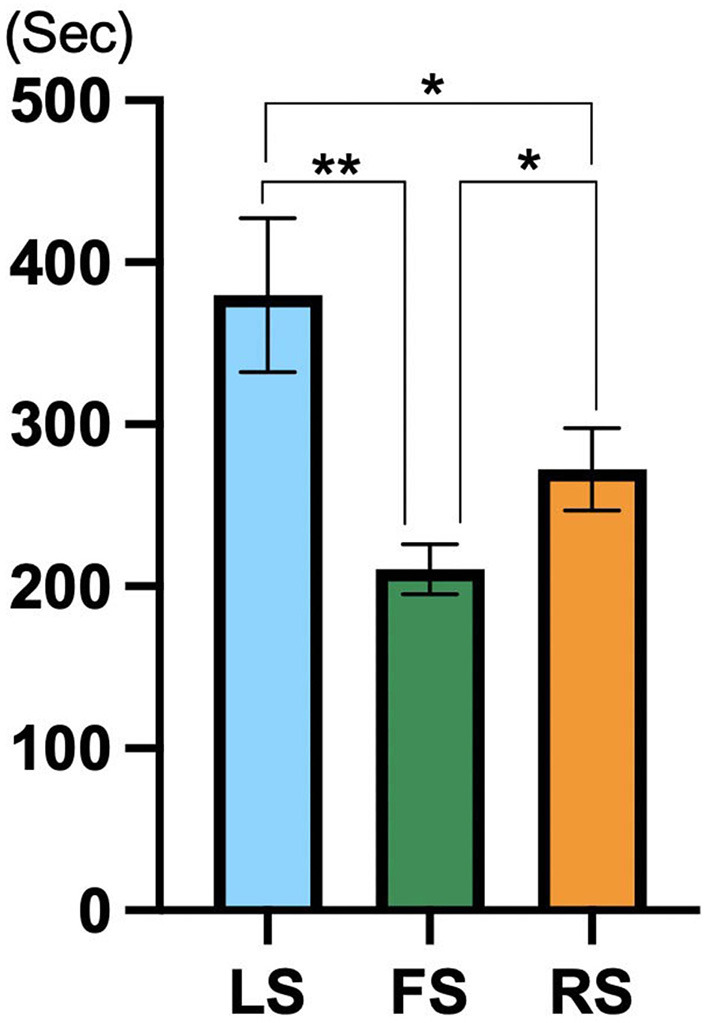
Comparison results of observation duration across the LS, FS, and RS (*N* = 30). ^*^p < 0.05, ^**^p < 0.01; ^***^p ≤ 0.001.

As shown in Table 1, the sites of the three experimental scenes significantly affected the observation duration. As shown in Figure 4, the observation duration for LS was significantly longer than that for FS [LS: 379.835 ± 47.528 vs. FS: 210.656 ± 15.284, 95% CI (65.638, 272.719), *p* = 0.001, *ηp*^2^ = 0.165, 1-β = 0.915] and RS [LS: 379.835 ± 47.528 vs. RS: 272.157 ± 25.450, 95% CI (11.786, 203.569), *p* = 0.024, *ηp*^2^ = 0.031, 1-β = 0.302], and the observation duration for RS was significantly longer than for FS [RS: 272.157 ± 25.450 vs. FS: 210.656 ± 15.284, 95% CI (20.325, 102.676), *p* = 0.043, *ηp*^2^ = 0.069, 1-β = 0.531].

**Table 1:** Statistical results for various data in the LS, FS, and RS (*N* = *30*).

**Category**	**Variable**	**LS**				**FS**				**RS**				**Comparison results**			
				**95% CI**				**95% CI**				**95% CI**					
		**Mean**	**Std. error**	**LB**	**UB**	**Mean**	**Std. error**	**LB**	**UB**	**Mean**	**Std. error**	**LB**	**UB**	*F*_(2, 28)_	***p***	*η**p2***	**1 -** β
Observation	Duration	379.835	47.528	282.630	477.041	210.657	15.284	179.398	241.915	272.158	25.450	220.106	324.209	11.114	0.000	0.443	0.985
POMS	AH	−0.467	0.202	−0.880	−0.054	0.333	0.393	−0.471	1.138	−0.267	0.143	−0.560	0.026	1.353	0.275	0.088	0.267
	CB	−1.700	0.429	−2.578	−0.822	0.067	0.510	−0.975	1.109	−0.200	0.373	−0.962	0.562	4.514	0.020	0.244	0.723
	DD	−1.467	0.383	−2.250	−0.684	1.067	0.374	0.301	1.832	−0.400	0.252	−0.915	0.115	11.992	0.000	0.461	0.990
	FI	−2.067	0.362	−2.807	−1.327	0.300	0.375	−0.467	1.067	0.000	0.498	−1.019	1.019	10.438	0.000	0.427	0.979
	TA	−1.567	0.454	−2.495	−0.638	0.433	0.491	−0.570	1.437	−0.300	0.362	−1.041	0.441	5.899	0.007	0.296	0.838
	VA	−2.733	0.705	−4.174	−1.292	−0.200	0.570	−1.366	0.966	0.333	0.501	−0.692	1.359	9.918	0.001	0.415	0.973
	F	−2.233	0.509	−3.274	−1.192	0.600	0.611	−0.650	1.850	0.500	0.575	−0.676	1.676	6.525	0.005	0.318	0.875
Questionnaire	1	3.867	0.190	3.478	4.256	3.000	0.166	2.660	3.340	2.867	0.224	2.410	3.324	12.174	0.000	0.465	0.991
	2	2.767	0.202	2.354	3.179	2.767	0.213	2.332	3.202	2.600	0.218	2.155	3.045	0.203	0.818	0.078	0.014
	3	3.500	0.157	3.178	3.822	3.167	0.204	2.750	3.584	3.067	0.219	2.618	3.515	2.155	0.135	0.133	0.404
	4	2.967	0.182	2.593	3.340	2.333	0.168	1.989	2.678	2.333	0.205	1.913	2.753	3.717	0.037	0.210	0.633
	5	4.100	0.154	3.785	4.415	2.533	0.190	2.144	2.922	3.867	0.196	3.466	4.268	20.939	0.000	0.599	1.000
	6	4.033	0.169	3.887	4.380	2.233	0.266	1.690	2.777	2.000	0.144	1.706	2.294	34.018	0.000	0.708	1.000
	7	3.467	0.196	3.066	3.868	1.933	0.151	1.624	2.242	3.667	0.154	3.351	3.982	55.302	0.000	0.798	1.000
	8	2.867	0.157	2.545	3.188	2.167	0.180	1.799	2.535	2.367	0.189	1.981	2.753	6.188	0.006	0.307	0.856

#### 3.1.2 Heart rate

**Figure 5:**
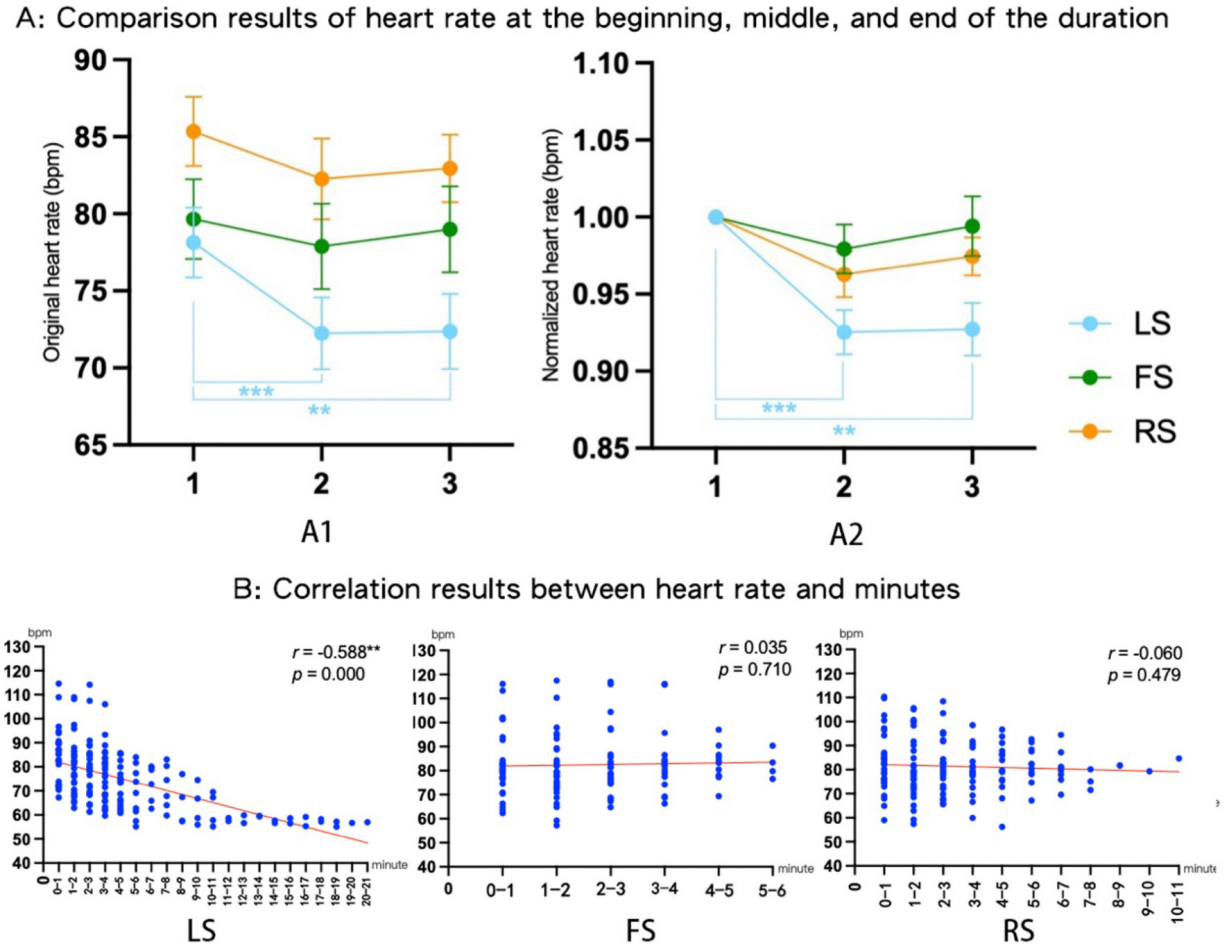
Heart rate results for different time periods in LS, FS, and RS [**(A)** *N* = 30, 1 represents the beginning of the duration, 2 represents the middle of the duration, and 3 represents the end of the duration. **(B)** The correlation results between heart rate and minutes]. ^*^p < 0.05, ^**^p < 0.01; ^***^p ≤ 0.001.

As shown in Table 2, the three time periods (the beginning, middle, and end) significantly affected the original average heart rate in LS, while they significantly affected the normalized average heart rate in both LS and RS. As shown in Figure 5A, in LS, the average heart rate during the last 30 s of the experiment was significantly lower than the first 30 s [the end: 72.360 ± 2.432 vs. the beginning: 78.129 ± 2.276, 95% CI (−2.363, −9.175), *p* = 0.001, *ηp*^2^ = 0.049, 1-β = 0.399], and the average heart rate during the middle 30 s was also significantly lower than that during the first 30 s [the middle: 72.245 ± 2.336 vs. the beginning: 78.129 ± 2.276, 95% CI (−8.697, −3.071), *p* = 0.000, *ηp*^2^ = 0.053, 1-β = 0.426]. Even after the heart rate was normalized (Figure 5A), the average heart rates during the last [the end: 0.927 ± 0.017 vs. the beginning: 1 ± 0, 95% CI (−0.116, −0.029), *p* = 0.001, *ηp*^2^ = 0.238, 1-β = 0.987] and middle 30 s [the middle: 0.925 ± 0.014 vs. the beginning: 1 ± 0, 95% CI (−0.111, −0.038), *p* = 0.000, *ηp*^2^ = 0.320, 1-β = 0.999] remained significantly lower than that during the first 30 s.

**Table 2:** Statistical results for heart rate at the beginning of the duration, the middle of the duration, and the end of the duration (each duration is 30 s, *N* = *30*).

**Measurement**	**Site**	**The beginning of the duration**				**The middle of the duration**				**The end of the duration**				**Comparison results**			
				**95% CI**				**95% CI**				**95% CI**					
		**Mean**	**Std. error**	**LB**	**UB**	**Mean**	**Std. error**	**LB**	**UB**	**Mean**	**Std. error**	**LB**	**UB**	**F**_(2, 28)_	***p***	*η**p2***	**1 -** β
Original heart rate	LS	78.129	2.276	73.474	82.783	72.245	2.336	67.467	77.023	72.360	2.432	67.385	77.334	13.820	0.000	0.497	0.996
	FS	79.662	2.585	74.376	84.948	77.882	2.771	72.214	83.551	78.987	2.792	73.278	84.697	0.994	0.383	0.066	0.205
	RS	85.352	2.262	80.725	89.979	82.263	2.635	76.874	87.652	82.954	2.192	78.470	87.437	3.257	0.053	0.189	0.572
Normalized heart rate	LS	1.000	0.000	1.000	1.000	0.925	0.014	0.896	0.955	0.927	0.017	0.892	0.962	13.352	0.000	0.488	0.995
	FS	1.000	0.000	1.000	1.000	0.979	0.016	0.947	1.012	0.994	0.019	0.954	1.034	0.899	0.419	0.060	0.189
	RS	1.000	0.000	1.000	1.000	0.963	0.015	0.932	0.993	0.974	0.012	0.950	0.999	3.373	0.049	0.194	0.588

#### 3.1.3 Profile of mood states, 2nd edition–adult (POMS 2–A)

**Figure 6:**
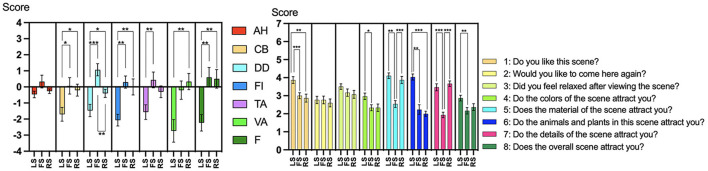
Comparison results of POMS and rated questions across the LS, FS, and RS (*N* = 30). ^*^p < 0.05, ^**^p < 0.01; ^***^p ≤ 0.001.

As shown in Table 1, with the exception of AH, the sites of the three experimental scenes significantly affected the other six mood states of the POMS. Lower values indicated better improvement in mood states (Figure 6).

The values of LS in CB [LS: −1.700 ± 0.429 vs. FS: 0.066 ± 0.510, 95% CI (−3.400, −0.134), *p* = 0.031, *ηp*^2^ = 0.053, 1-β = 0.428], DD [LS: −1.466 ± 0.383 vs. FS: 1.066 ± 0.374, 95% CI (−3.866, −1.200), *p* = 0.000, *ηp*^2^ = 0.279, 1-β = 0.996], FI [LS: −2.066 ± 0.362 vs. FS: 0.300 ± 0.375, 95% CI (−3.769, −0.964), *p* = 0.001, *ηp*^2^ = 0.262, 1-β = 0.994], TA [LS: −1.566 ± 0.454 vs. FS: 0.433 ± 0.491, 95% CI (−3.492, −0.508), *p* = 0.006, *ηp*^2^ = 0.134, 1-β = 0.837], and F [LS: −2.233 ± 0.509 vs. FS: 0.600 ± 0.611, 95% CI (−4.887, −0.780), *p* = 0.001, *ηp*^2^ = 0.179, 1-β = 0.938] were all significantly lower than those of FS.

The values of LS in CB [LS: −1.700 ± 0.429 vs. RS: −0.200 ± 0.373, 95% CI (−2.898, −0.102), *p* = 0.032, *ηp*^2^ = 0.011, 1-β = 0.125], DD [LS: −1.466 ± 0.383 vs. RS: −0.400 ± 0.252, 95% CI (−2.010, −0.124), *p* = 0.023, *ηp*^2^ = 0.085, 1-β = 0.629], FI [LS: −2.066 ± 0.362 vs. RS: 0.000 ± 0.498, 95% CI (−3.492, −0.641), *p* = 0.003, *ηp*^2^ = 0.163, 1-β = 0.910], VA [LS: −2.733 ± 0.705 vs. RS: 0.333 ± 0.501, 95% CI (−5.109, −1.025), *p* = 0.002, *ηp*^2^ = 0.178, 1-β = 0.937], and F [LS: −2.233 ± 0.509 vs. RS: 0.500 ± 0.575, 95% CI (−4.786, −0.681), *p* = 0.002, *ηp*^2^ = 0.177, 1-β = 0.937] were all significantly lower than those of RS. The RS value was significantly lower in DD than in FS [RS: −0.400 ± 0.252 vs. FS: 1.066 ± 0.374, 95% CI (−2.334, −0.599), *p* = 0.001, *ηp*^2^ = 0.154, 1-β = 0.892].

The Chinese version of POMS 2-A demonstrated good psychometric properties in the current sample. Internal consistency reliability coefficients (Cronbach's α) for the seven subscales were as follows: Anger-Hostility (α = 0.82), Confusion-Bewilderment (α = 0.79), Depression-Dejection (α = 0.85), Fatigue-Inertia (α = 0.81), Tension-Anxiety (α = 0.83), Vigor-Activity (α = 0.78), and Friendliness (α = 0.76), indicating acceptable to good reliability for all subscales.

Confirmatory factor analysis supported the seven-factor structure of POMS 2-A, with the model showing adequate fit to the data: χ^2^/df = 2.36, CFI = 0.92, RMSEA = 0.07 (90% CI: 0.06–0.08), SRMR = 0.06. All items loaded significantly on their respective factors (standardized factor loadings ranged from 0.52 to 0.81), providing evidence for the structural validity of the measure.

Criterion validity was supported by significant correlations between POMS subscale scores and heart rate changes. Greater reductions in heart rate were associated with greater improvements in negative mood states (DD: *r* = −0.699, *p* = 0.005).

#### 3.1.4 Rated questions of the supplemental questionnaire

As shown in Table 1, except for the second and third questions, the sites of the three experimental scenes significantly affected the other six rated questions of the Supplemental Questionnaire. Figure 6 displays the comparison of the average scores for each rated question across LS, FS, and RS for the 30 participants.

In the first [LS: 3.867 ± 0.190 vs. FS: 3.000 ± 0.166, 95% CI (0.383, 1.350), *p* = 0.000, *ηp*^2^ = 0.622, 1-β = 1.000], fourth [LS: 2.967 ± 0.182 vs. FS: 2.333 ± 0.168, 95% CI (0.018, 1.248), *p* = 0.013, *ηp*^2^ = 0.101, 1-β = 0.708], fifth [LS: 4.100 ± 0.154 vs. FS: 2.533 ± 0.190, 95% CI (0.881, 2.252), *p* = 0.002, *ηp*^2^ = 0.414, 1-β = 1.000], sixth [4.033 ± 0.169 vs. FS: 2.233 ± 0.266, 95% CI (1.250, 2.350), *p* = 0.001, *ηp*^2^ = 0.398, 1-β = 1.000], seventh [LS: 3.467 ± 0.196 vs. FS: 1.933 ± 0.151, 95% CI (0.979, 2.088), *p* = 0.000, *ηp*^2^ = 0.398, 1-β = 1.000], and eighth [LS: 2.867 ± 0.157 vs. FS: 2.167 ± 0.180, 95% CI (0.195, 1.205), *p* = 0.005, *ηp*^2^ = 0.129, 1-β = 0.822] rated questions, the average LS score was significantly higher than the FS score.

In the first [LS: 3.867 ± 0.190 vs. RS: 2.867 ± 0.224, 95% CI (0.333, 1.667), *p* = 0.002, *ηp*^2^ = 0.593, 1-β = 1.000] and sixth [LS: 4.033 ± 0.169 vs. RS: 2.000 ± 0.144, 95% CI (1.406, 2.660), *p* = 0.000, *ηp*^2^ = 0.591, 1-β = 1.000] rated questions, the average LS score was significantly higher than the RS score.

In the fifth [RS: 3.867 ± 0.196 vs. FS: 2.533 ± 0.190, 95% CI (0.720, 1.947), *p* = 0.000, *ηp*^2^ = 0.291, 1-β = 0.998] and seventh [RS: 3.667 ± 0.154 vs. FS: 1.933 ± 0.151, 95% CI (1.279, 2.188), *p* = 0.000, *ηp*^2^ = 0.526, 1-β = 1.000] rated questions, the average RS score was significantly higher than the FS score.

#### 3.1.5 Results of the correlation analysis

The correlation analysis results for observation duration and heart rate or POMS are presented in Figure 5B and Table 3. Among the three scenes, only LS showed a significant correlation, where the observation duration showed a moderately negative correlation with heart rate, suggesting that a longer duration was associated with a greater reduction in heart rate. However, Figure 5B shows that the heart rate plateaued after 11 min. To examine the influence of post-11-min data on the overall correlation, the data were divided into Group 1 (before 11 min) and Group 2 (after 11 min), and Fisher's Z-transformation was used to test for significant differences in the correlation between the two groups. The results revealed no significant differences (*Z* = 0.042, *p* = 0.966) in correlation between Group 1 (*r* = −0.463^**^, *p* = 0.000) and Group 2 (*r* = −0.472, *p* = 0.065), thereby demonstrating that the overall significant negative correlation trend was not significantly affected by the post-11-min data.

**Table 3:** Partial correlation between observation duration and POMS, controlling for order (Group A, Group B, and Group C) in Experiment 1 (*N* = *30*).

**Site**	**Variable**	**Statistic**	**AH**	**CB**	**DD**	**FI**	**TA**	**VA**	**F**
LS	Observation duration	*r*	−0.124	−0.499^**^	−0.699^**^	−0.167	−0.268	0.13	−0.11
		*p*	0.963	0.515	0.005	0.377	0.366	0.152	0.493
	Rated question	*r*	−0.420^*^	−0.385^*^	0.049	−0.055	−0.401^*^	0.382^*^	−0.381^*^
		*p*	0.021	0.036	0.795	0.775	0.028	0.037	0.038
FS	Observation duration	*r*	0.012	0.089	−0.277	−0.178	−0.069	−0.242	−0.164
		*p*	0.952	0.642	0.138	0.346	0.718	0.198	0.386
	Rated question	*r*	0.141	0.136	−0.383^*^	0.426^*^	−0.113	0.396^*^	−0.226
		*p*	0.458	0.474	0.036	0.019	0.553	0.03	0.229
RS	Observation duration	*r*	−0.239	−0.042	−0.087	−0.224	−0.315	−0.302	−0.377^*^
		*p*	0.203	0.824	0.647	0.233	0.09	0.105	0.04
	Rated question	*r*	−0.388^*^	0.399^*^	0.390^*^	−0.206	−0.428^*^	−0.376^*^	−0.242
		*p*	0.034	0.029	0.033	0.274	0.018	0.041	0.199

^*^p < 0.05, ^**^p < 0.01; ^***^p ≤ 0.001.

Additionally, in LS, the observation duration showed a moderately negative correlation with DD, suggesting that a longer duration was associated with a greater reduction in negative emotions and a greater sense of relaxation. Furthermore, the sum of the rated questions showed a significant negative correlation with CB and TA.

#### 3.1.6 Comparative summary between experiment 1 and experiment 2

After all the data results were obtained, a summary table integrating the results of Experiment 1 and Experiment 2 was created (Table 4) to improve the readability and clarity of the entire study.

**Table 4:** Comparative summary of key findings from Experiment 1 and Experiment 2.

**Category**	**Experiment 1 (Landscape types)**	**Experiment 2 (Poetry task)**
Primary focus	Comparison of relaxation effects among water (LS), forest (FS), and rock (RS) scenes	Additional effects of poetry composition during water scene (LS) viewing (NP vs. WP)
Observation duration	LS>RS>FS (LS:379.8 s, RS:272.2 s, FS:210.7 s; *p* < 0.05)	NP>WP (NP:337.8 s, WP:216.2 s; *p* = 0.000, Cohen's *d* = 0.908)
Heart rate reduction	Significant reduction only in LS (72.4 vs. baseline 78.1 bpm; *p* = 0.001)	No NP-WP difference (*p* =0.152), but stronger HR duration correlation in WP(*r* = −0.53)
Psychological effects	LS significantly improved negative moods (CB, DD, FI, TA; *p* < 0.05)	WP group (16/30 participants) showed deeper reflection (memories, philosophy) with higher poem quality scores (*d* =1.51)
Key mechanism	Water's visual appeal and spatial composition prolonged viewing and promoted relaxation	Poetry enhanced cognitive engagement through nature interaction, despite shorter viewing times
Limitations	Sample limited to Chinese Han Buddhism practitioners, and only investigated the meditation landscape of one Buddhist temple	Fixed 5-line format may constrain creative expression. More poetic forms are needed to verify the broad applicability of this behavior

### 3.2 Discussion

Although the pavilions (Ting) built for viewing nature reflect the influence of “the unity of human and nature” on Chinese Buddhism, this study empirically examined Chinese Buddhist natural landscapes and found that while monks chose diverse natural settings for contemplation, the physiological and psychological effects varied significantly with the differences in landscape composition.

In terms of physiological responses, only viewing LS significantly reduced the heart rate among the three scenes (Figure 5A), and this effect improved with longer viewing durations, stabilizing at around 60 bpm after 11 min (Figure 5B). Given that the resting human heart rate typically ranges between 60 and 100 bpm (Olshansky et al., 2023) and a lower heart rate indicates relaxation (Pfister et al., 2012), after 11 min of LS viewing, the participants' heart rates stabilized at the lower limit of the resting range, signifying the attainment of an optimal physiological relaxed state. We posit that these divergent trends stem from the distinct visual experiences provided by each scenario, given that over 80% of sensory input is visually processed (Porteous, 2013). Based on these findings and perspectives, we focused on analyzing the landscape characteristics of the three scenes to investigate the underlying causes of the physiological response differences.

**Figure 7:**
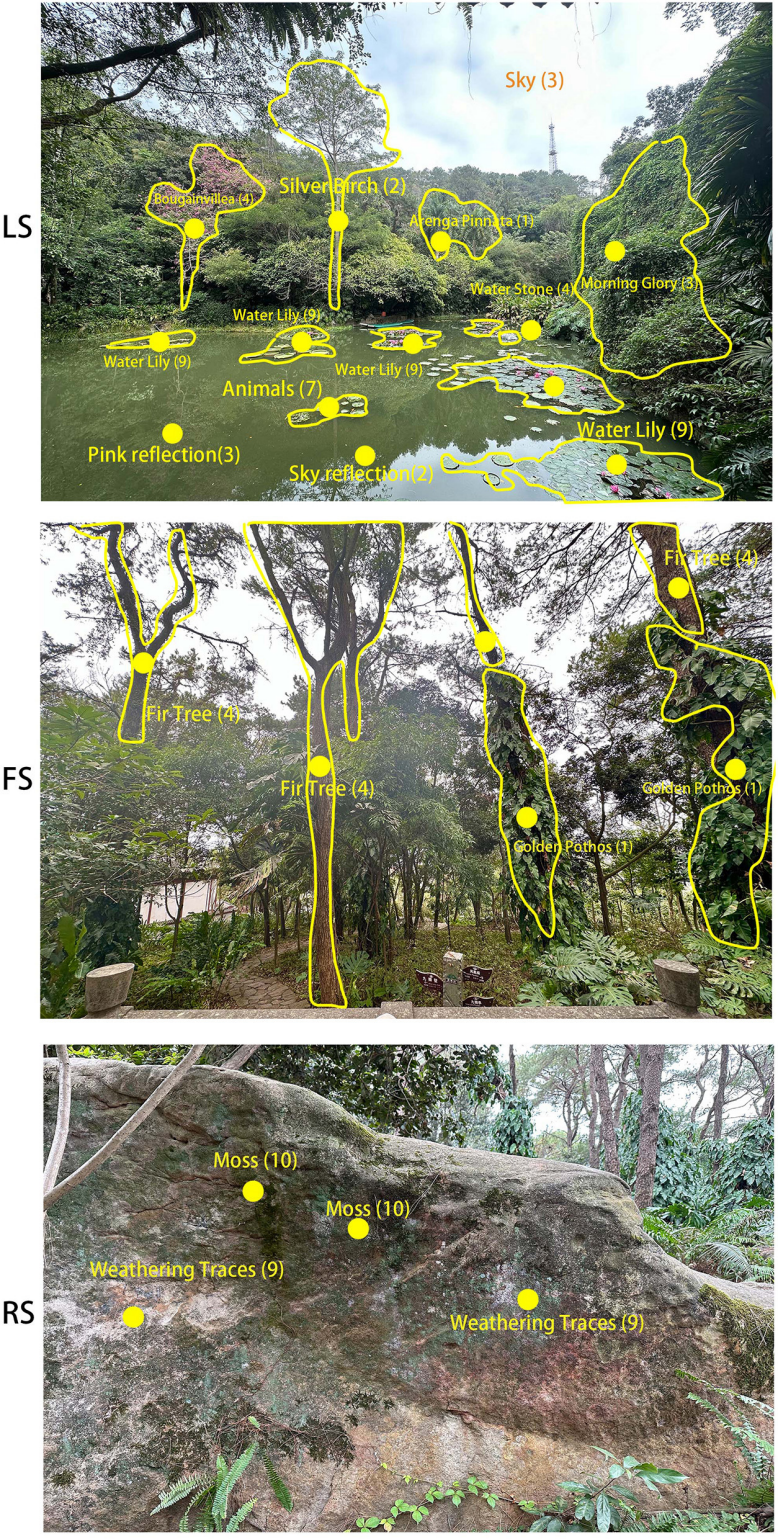
The frequency of landscape objects mentioned by 30 participants in post-experimental comments across LS, FS, and RS (the numbers in parentheses indicate the frequency of mentions).

From a compositional perspective, the LS features an expansive layout with clearly delineated foreground, middle ground, and background zones. This well-structured spatial arrangement likely reduces visual cognitive load caused by complex configurations. The results of the mentioned observed objects showed that, compared to other scenes, the landscape elements mentioned in the LS were widely distributed across the scene, and even objects located at the visual periphery (such as *Bougainvillea* and *Morning Glory*) or those that were smaller (such as Water Stones) still attracted the participants' attention (Figure 7). Therefore, the participants observed a broader range of features in the LS and were more easily able to recognize their positions, supporting the aforementioned hypothesis. The research found that layered open landscapes increase environmental detail salience, aiding object recognition and inducing relaxation (Liang et al., 2024). Therefore, it can be inferred that the overall composition in LS has a dual effect: it enhances the desire for visual exploration and promotes progressive physiological relaxation (Figure 5B).

Furthermore, post-experiment feedback from LS participants specifically highlighted how the water landscape deepened their engagement and relaxation. In previous studies, analyzing participants‘ visual perception of landscapes using their free comment feedback has demonstrated objective validity (Elsadek et al., 2019; Goto et al., 2017, 2020; Liang et al., 2024; Sun et al., 2018). Therefore, it is reasonable to use the post-experiment comments from Experiment 1 to evaluate the participants' perceptions. Representative comments for LS included, “The reflections on the lake surface drew me into deep immersion, allowing me to naturally regulate my breathing rhythm,” and “The mirror-like water surface left a lasting impression, creating a profound sense of tranquility.” Empirical research confirmed that water reflections significantly enhanced people's appreciation of the scene (Sun and Bao, 2025). These findings demonstrate that the well-arranged spatial composition coupled with the inherent attributes of the water landscape collectively contribute to superior overall landscape quality in LS. In this experiment, this integrated quality showed the potential to enhance participant engagement in landscape viewing; thus, prolonged visual pleasantness ultimately ensures consistent psychological relaxation effects, as evidenced by both the behavioral (prolonged observation duration) and physiological (progressive heart rate reduction) measurements (Figure 5B).

In contrast, the FS featured a more constrained view, as the background was obscured by trees. The middle ground, separated by vertical blocks of foliage, limits the visual depth, creating a more fragmented landscape. Disorganized scene compositions tend to increase visual load (Milam et al., 2011), so the FS's cluttered structure may have hindered effortless perception and reduced the viewer's sense of immersion. Moreover, the dense tree cover in FS also resulted in dimmer lighting, obstructing the participants' ability to discern finer details within the scene, diminishing the clarity and depth of observation essential for deep landscape meditation. These factors were not conducive to sustained physiological relaxation, which may explain why participants did not experience a significant decrease in heart rate (Figure 5).

In the case of RS, the close-distant viewing allowed the participants to carefully observe the details of the rocks. Some participants provided detailed comments on the rock's features, with key observations including “unique textures,” “tiny yet resilient moss showcasing tenacious vitality,” and “the white veins captivated my lingering gaze.” These evaluations indicate that the closer distance viewing in RS successfully captured the participants‘ sustained attention; if a stimulus captures attention, one might also anticipate increased sensory intake accompanied by a noticeable decrease in heart rate (Laumann et al., 2003). However, eight participants described RS as merely a “boring rock,” reflecting dissatisfaction with the scene's monochromatic material. This suggests that while close observation may encourage detailed scrutiny, the lack of material diversity may hinder sustained attention. This effect might indirectly manifest in participants' physiological responses, resulting in no significant reduction in heart rate (Figure 5).

In terms of psychological responses, LS was most effective at improving mood states, as evidenced by the POMS measures (Figure 6), and this effect tended to improve with increasing duration (Table 3). These findings align with prior research highlighting the importance of passive engagement with natural stimuli for emotional regulation. For instance, Nisbet et al. (2019) found that in natural settings, meditation interventions requiring intentional attention control showed no significant positive emotional effects over passive exposure. This lack of a pronounced difference may indicate the need for further investigation into interventions involving intentional attention control, while also potentially being attributed to whether the specific characteristics of the natural environment are conducive to sustaining participants‘ long-term interest and engagement. Although Experiment 1 did not require the participants to intentionally control their attention, its findings still suggest a potential mechanism: if the characteristics of natural stimuli employed in meditation practice align with participants' preferences and effectively engage their interest, nature-based interventions may play a dominant role in enhancing relaxation of emotional states for meditation practice.

The results of the rated questions and correlation analysis corroborated this hypothesis. Firstly, participants‘ evaluations revealed that they preferred LS the most, with its landscape characteristics receiving significantly higher ratings than those of other scenes (Figure 6). Additionally, LS had the highest number of landscape objects mentioned by participants (Figure 7). These findings indicate that the landscape objects in LS were more attention-grabbing. Secondly, Table 3 revealed that higher LS rating scores correlated with improvements in two mood states. This pattern suggests that participants' degree of landscape preference directly correlates with psychological benefits, with characteristics of LS emerging as the most preferred and psychologically beneficial environment, further confirming, from the perspective of psychological response, the effectiveness of LS's landscape quality and visual attractiveness in producing positive outcomes.

However, while viewing distant LS yielded more positive effects and better evaluations than close-range RS, the opposite pattern was observed between RS and distant FS (Figures 4–6). This contradictory phenomenon can be attributed to not only distance but also the participants' visual habits and the level of attraction of the scene (Chamberlain, 2007; Li et al., 2018; Sun and Bao, 2025). When uncertain about where to direct their gaze, humans tend to focus straight ahead (Rothkegel et al., 2017; Yarbus, 2013). The most prominent objects directly in front of the FS were several tall fir trees, which were unavoidable. Eight participants expressed dissatisfaction with the plants in FS, describing them as “monotonous,” “cluttered,” “unappealing,” and too close together, creating a “sense of oppression” and a desire for “more open space.” It can be inferred that these fir trees were passively observed rather than preferred. Similar to the findings of this study, a previous study found that distant plants may fail to draw visual attention (possibly processed unconsciously), while nearby rocks attract focused attention, likely due to interest in their surface details (Liang et al., 2024). Therefore, focusing on proximate features enhances engagement more effectively than viewing less appealing distant scenes. On the other hand, while the distant LS scene lacked the close-up visual experience of RS, it offered a wide, clear view through high-quality landscapes, reducing cognitive load from unattractive elements and offsetting the impact of distance. In contrast, FS, with a similar viewing distance to LS, had complex, chaotic layers that could cause visual overload, making it difficult for individuals to relax. While this study suggests that the effects of observing nature are influenced by both landscape quality and distance, these effects also vary depending on the balance between these factors.

In summary, this study found that viewing nature-integrated environments, as advocated by Taoism, encourages longer viewing periods and induces a more relaxing effect than gazing at rocks—as originally practiced by the Indian monk Bodhidharma (Du and Wei, 1993). Lymeus et al. (2017) showed that nature images reduce meditation-induced attentional strain, enhancing psychological recovery. In contrast, this study used real natural settings, demonstrating positive effects based on landscape quality and visibility, deepening our understanding of how specific nature characteristics promote relaxation. As evidenced in Experiment 1, the water landscape captured more attention owing to its visually engaging properties, leading to longer observation times. As participants' observations deepened, they made more new discoveries, and further reflection ensued, accompanied by better relaxation effects. These findings suggest that the water landscape allowed participants to immerse themselves more fully in nature, promoting a deeper restorative experience through dynamic visual stimuli and continuous sensory engagement, which static elements like rocks cannot provide. While these findings originate from philosophical Buddhist practices, empirical research may offer scientifically meaningful insights into contemporary real-world issues.

## 4 Experiment 2

### 4.1 Results

The results from Experiment 1 were input into the formula for calculation, yielding the following results:

For LS, the final score = 379.835 (observation duration) ^*^ 40% + (−0.927) (normalized heart rate) ^*^ 40% + 27.566 (rated questions) ^*^ 20% = 157.0764; For FS, the final score = 210.656 (observation duration) ^*^ 40% + (−0.994) (normalized heart rate) ^*^ 40% + 21.700 (rated questions) ^*^ 20% = 88.2048; For RS, the final score = 272.157 (observation duration) ^*^ 40% + (−0.974) (normalized heart rate) ^*^ 40% + 22.766 (rated questions) ^*^ 20% = 113.0264.

Sensitivity analysis confirmed that the water scene (LS) consistently ranked highest across all weight schemes (Table 5), supporting the robustness of our original 40-40-20 distribution. Minor score variations did not alter the conclusion that LS was optimal for Experiment 2.

**Table 5:** Sensitivity analysis of final score weighting schemes.

**Weight scheme**	**LS**	**FS**	**RS**	**Top-ranked scene**
Original (40-40-20)	157.08	88.2	113.03	LS
Equal (33-33-33)	151.92	85.1	110.45	LS
Physiological (50-30-20)	159.15	86.75	115.62	LS
Behavioral (30-50-20)	154.33	89.4	108.91	LS

Since LS had the highest final score, it was selected as the site for Experiment 2.

#### 4.1.1 Observation duration and heart rate

The results in Table 6 show that the average observation duration for the 30 participants in the NP experiment was significantly higher than that in the WP experiment, with no significant difference in heart rate between the NP and WP experiments.

**Table 6:** The results of the paired-sample *t*-test for observation duration and heart rate in the experimental NP and WP (*N* = *30*).

**Measurement**	**Variable**	**NP**				**WP**				**Comparison results**		
				**95% CI**				**95% CI**				
		**Mean**	**Std. error**	**LB**	**UB**	**Mean**	**Std. error**	**LB**	**UB**	**t (29)**	***p***	***d***
Observation	Duration	337.815	21.627	296.046	376.771	216.168	18.473	180.036	253.124	4.971	0.000	0.908
HR	The first 30 s	79.967	2.659	75.051	84.817	79.028	2.018	75.352	82.598	0.267	0.792	0.049
	The middle 30 s	79.507	2.525	74.982	84.449	80.083	2.344	75.603	84.523	−0.149	0.882	−0.027
	The last 30 s	76.640	2.326	72.260	80.946	77.652	2.035	77.778	85.294	−1.471	0.152	−0.268
Normalized HR	The first 30 s	ns	ns	ns	ns	ns	ns	ns	ns	ns	ns	ns
	The middle 30 s	0.998	0.015	0.972	1.027	1.016	0.020	0.979	1.058	−0.699	0.490	−0.128
	The last 30 s	0.963	0.014	0.937	0.987	0.982	0.019	0.991	1.069	−0.814	0.118	−0.119

#### 4.1.2 Key content of five-line poems in WP

**Figure 8:**
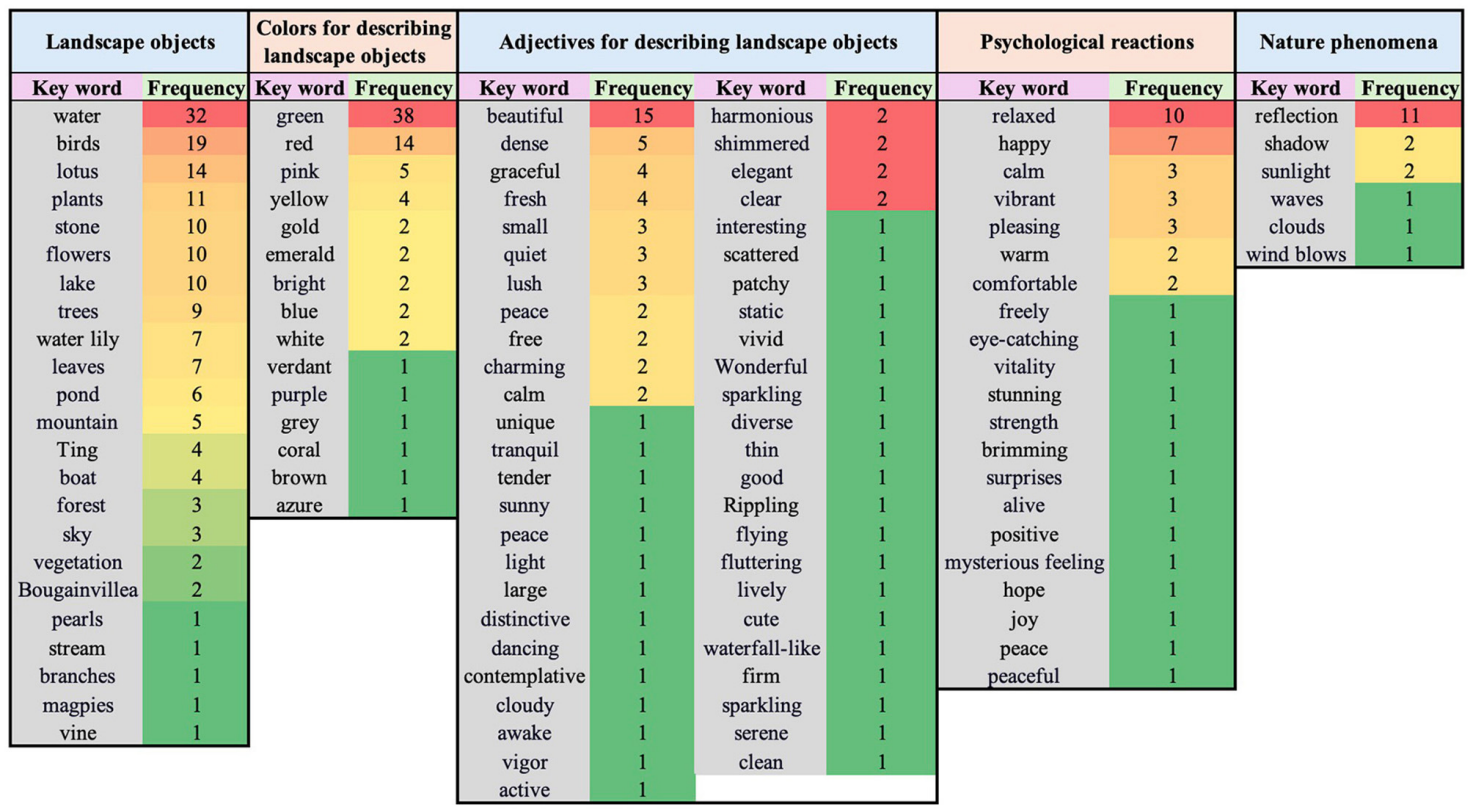
All keywords mentioned by the 30 participants across the five categories and their frequencies in WP.

Figure 8 displays all the keywords mentioned by the 30 participants in WP across the five categories and their frequencies. Among them, the average numbers of keywords mentioned per participant were as follows: 5.43 (± 1.813) under Landscape Objects, 2.57 (± 2.329) under Colors for Describing Landscape Objects, 2.90 (± 1.348) under Adjectives for Describing Landscape Objects, 1.47 (± 1.041) under Psychological Reactions, and 0.60 (± 0.563) under Nature Phenomena.

#### 4.1.3 Results of the correlation analysis

**Figure 9:**
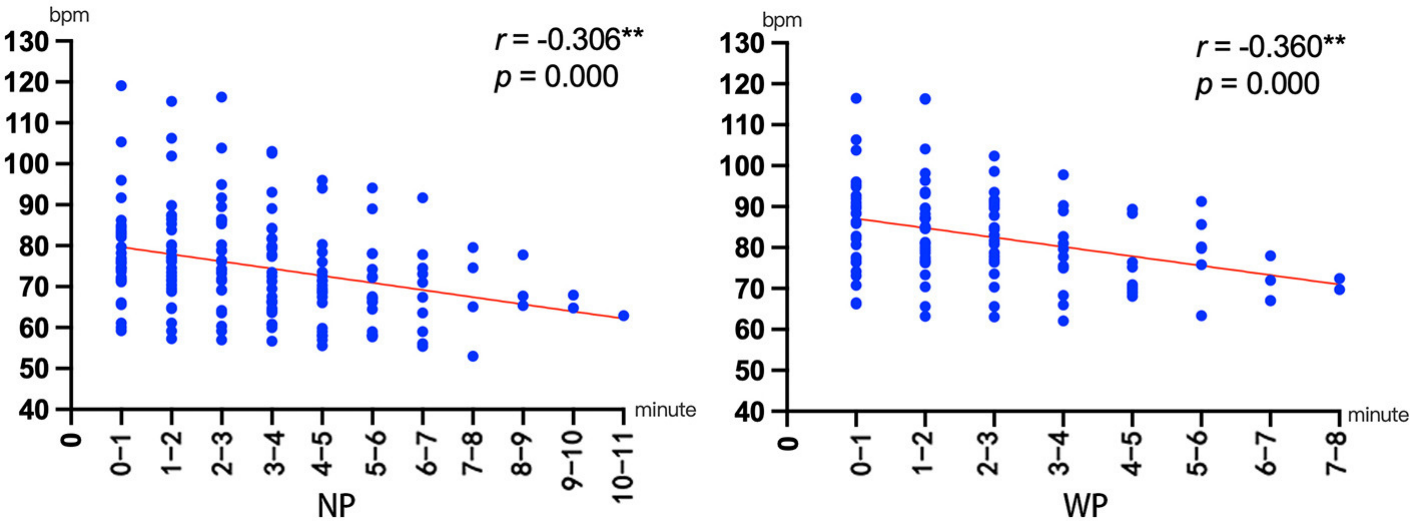
The correlation results between heart rate and minutes in Experiment 2. ^*^p < 0.05, ^**^p < 0.01; ^***^p ≤ 0.001.

Figure 9 shows that in both NP and WP, the observation duration and heart rate had a weak negative correlation, suggesting that a longer duration was associated with a greater reduction in heart rate.

#### 4.1.4 Results of analysis of participant data based on differences in poem content

**Figure 10:**
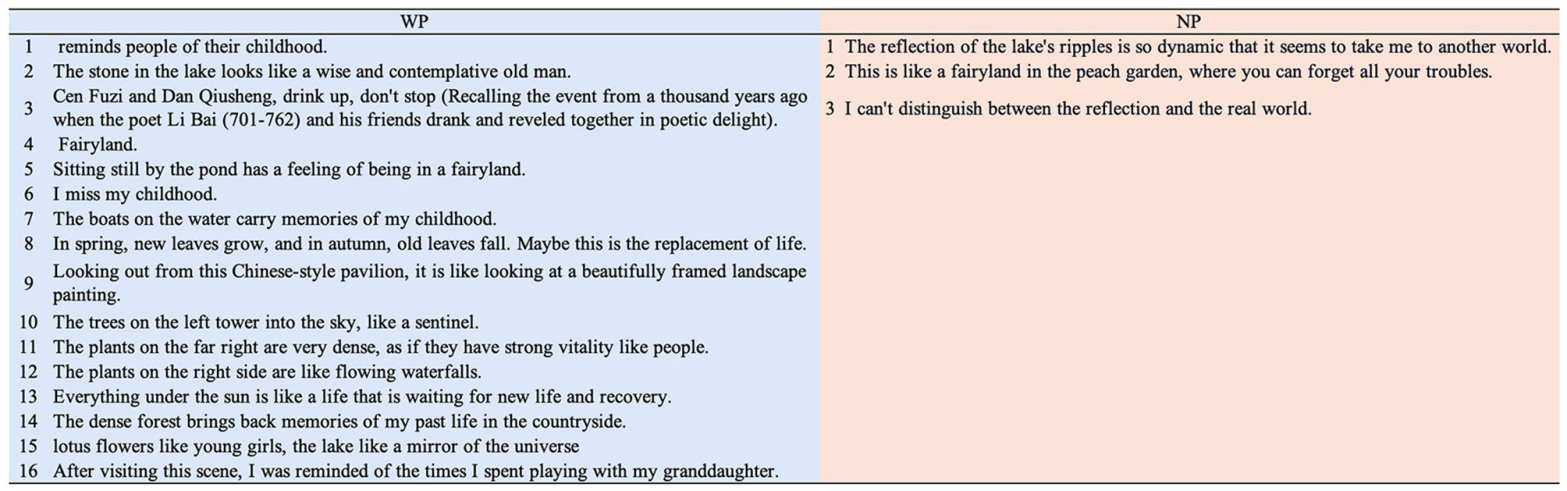
The personal thoughts beyond the landscape mentioned by participants in both the WP and NP experiments.

An in-depth investigation into the content of the poems in WP revealed that 16 participants discussed personal reflections beyond the landscape (Figure 10). Among the 16 participants in WP, six mentioned ideas related to memories, seven metaphorically compared the landscape in LS to other things, and three began to discuss the meaning of life.

**Figure 11:**
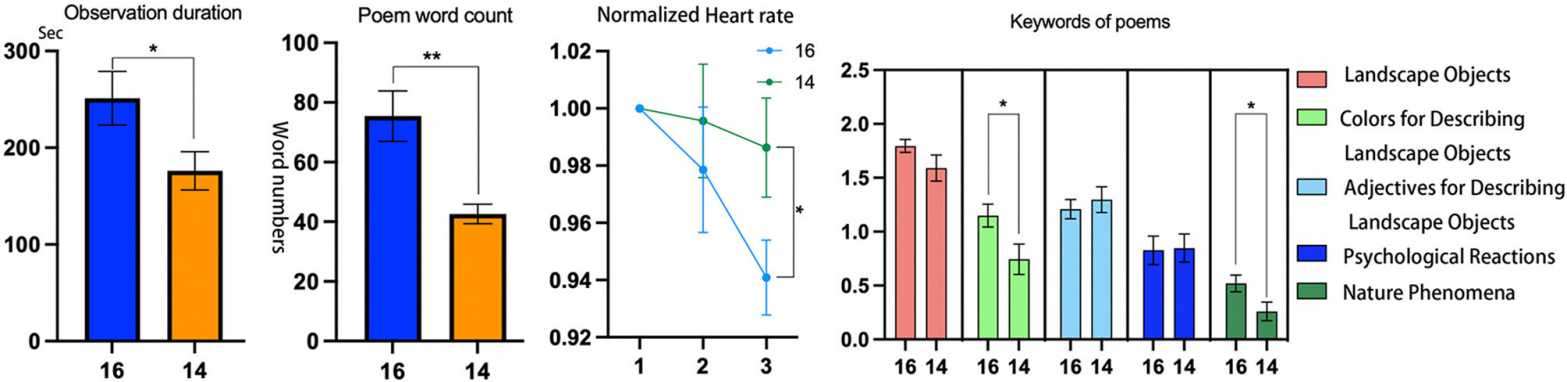
Comparison of the normalized heart rates, observation duration, and poetry content between 16 participants and 14 participants in the WP experiment (in heart rate results, 1 represents the beginning of the duration, 2 represents the middle of the duration, and 3 represents the end of the duration). ^*^p < 0.05, ^**^p < 0.01; ^***^p ≤ 0.001.

When comparing the data of these 16 participants with the other 14 participants who did not mention reflections beyond the landscape WP, it was found that the observation duration of the 16 participants was significantly longer [16: 251.183 ± 27.727 vs. 14: 176.150 ± 19.657, 95% CI (3.516, 146.551), *t* (28) = 2.149, *p* = 0.040, *d* = 0.786] (Figure 11), while the average normalized heart rate of the 16 participants was significantly lower than that of the 14 participants in the last 30 s of the experiment (Figure 11).

Additionally, in terms of the word count for poetry composition [16: 75.437 ± 8.457 vs. 14: 42.642 ± 3.247, 95% CI (13.223, 52.366), *t* (28) = 3.432, *p* = 0.002, *d* = 1.256] as well as the number of keywords for colors [16: 2.870 ± 0.092 vs. 14: 2.222 ± 0.163, 95% CI (0.051, 0.795), *t* (28) = 2.332, *p* = 0.027, *d* = 0.853] and natural phenomena [16: 0.744 ± 0.163 vs. 14: 0.297 ± 0.095, 95% CI (0.101, 0.792), *t* (28) = 2.650, *p* = 0.013, *d* = 0.970], the group of 16 participants showed significantly higher results (Figure 11).

#### 4.1.5 Poem quality assessment

The inter-rater reliability for poem evaluation was excellent, with ICCs ranging from 0.82 to 0.89 across the four dimensions [use of descriptive language: ICC = 0.85, 95% CI (0.79, 0.90); positive emotional expression: ICC = 0.82, 95% CI (0.75, 0.88); reflection depth: ICC = 0.87, 95% CI (0.82, 0.91); structural coherence: ICC = 0.89, 95% CI (0.84, 0.92)]. The final score for each dimension was calculated as the average of the three raters' scores.

Participants demonstrated good performance across all dimensions. The average scores were: use of descriptive language = 4.12 ± 0.32, positive emotional expression = 3.87 ± 0.41, reflection depth = 3.95 ± 0.38, and structural coherence = 4.23 ± 0.29. Participants who engaged in deeper reflection (*n* = 16) scored significantly higher on positive emotional expression [4.31 ± 0.28 vs. 3.32 ± 0.35, *t* (28) = 4.12, *p* < 0.001, *d* = 1.51] and reflection depth [4.25 ± 0.31 vs. 3.56 ± 0.33, *t* (28) = 3.78, *p* = 0.001, *d* = 1.38] compared to those who did not.

A multiple correlation analysis was conducted between the poetry content ratings and heart rate data for the 16 participants engaged in deeper reflection in the WP condition compared to the other 14 participants. The results revealed a significant, very strong negative correlation between heart rate and positive emotional expression (Table 7).

**Table 7:** Partial correlation between heart rate and the score of the poem quality assessment in the WP of Experiment 2, for the 16 participants who engaged in deeper reflection and the other 14 participants, controlling for order (Group A and Group B).

**Group**	**Variable**	**Statistic**	**Use of descriptive language**	**Positive emotional expression**	**Reflection depth**	**Structural coherence**
16 participants	HR	*r*	0.314	−0.829^**^	−0.189	−0.195
		*p*	0.237	0.000	0.484	0.470
14 participants	HR	*r*	0.186	0.197	0.501	0.408
		*p*	0.524	0.500	0.068	0.148

^*^p < 0.05, ^**^p < 0.01; ^***^p ≤ 0.001.

### 4.2 Discussion

Building on the results of Experiment 1, Experiment 2 further explored the effects of thinking about poetry while observing a water landscape. The results in Table 6 show that the observation duration was in WP shorter than in NP. This discrepancy may be related to the participants' background. In ancient China, many Buddhist monks were originally Confucian scholars who had mastered sophisticated classical poetry skills (Du and Wei, 1993). In contrast, most modern Chinese individuals are unfamiliar with these techniques, making them unable to employ poetry as skillfully as ancient monks did for mental cultivation during landscape meditation. Consequently, the participants might have terminated the experiment immediately after mentally composing five poetic lines, resulting in shorter observation times. In addition, as a preliminary exploration, this study was limited to one poetic form, where composing five verses may have caused psychological tension, restricting thought development and resulting in shorter duration. Future research should explore more effective or feasible poetic forms. Nevertheless, data analysis still confirmed WP's positive effects as well as the higher efficiency in the generation patterns of these effects.

In terms of physiological responses, the correlation analysis results revealed significantly negative correlations between observation duration and heart rate in both NP and WP (Figure 9). Notably, the WP condition exhibited a stronger negative correlation (with an *r*-value closer to −1) than NP. This suggests that if the observation duration in WP were intentionally extended, rather than left to the discretion of participants unfamiliar with Buddhist meditation traditions, WP might demonstrate greater potential in inducing better physiological relaxation effects.

In terms of psychological responses, the content of the poems reveals that the WP experiment enhanced the participants' psychological cognition. Although WP only required participants to mention three objects, they listed an average of 5.43 objects, which increased to 6.03 when including natural phenomena (Figure 8). Additionally, while the WP experiment did not limit how participants described landscape objects, they used various colors and adjectives. Despite the observation duration in WP being significantly shorter than in NP, these results suggest that participants did not merely aim to fulfill the task; the WP stimuli enhanced their further exploration. As a result, they produced content richer than the experimental requirements. Whether or not their intention was to elaborate the poetry, they engaged in a deeper recognition of the physical form of the landscape within a shorter time in WP, which was also reflected in their subjective emotional judgments.

Furthermore, 16 participants in WP (vs. 3 in NP) reflected beyond landscape features, demonstrating WP's greater capacity for deeper contemplation (Figure 10). These 16 individuals also showed deeper contemplative engagement and better physiological relaxation (Figure 11) than the other 14 WP participants. This suggests that the deeper the participants' thinking in the WP, the more relaxed they became. Among the 16 participants, the content of their memories included “playing with my granddaughter,” “childhood,” and “friendship.” In terms of imagination, many of them compared LS to the fairyland (Xian Jing) in Taoist philosophy, where Taoist immortals reside and enjoy a carefree and joyful life. Philosophical reflections on life, such as “replacement of life” and “life and recovery,” were also mentioned several times, as new life often brings hope and vitality (Cioran, 2013). These contents reflect the participants' comfortable emotional state. The correlation analysis further revealed that when these 16 participants demonstrated higher-quality positive emotional responses in their poems, their heart rates showed a significant decreasing trend (Table 7). This suggested that the deeper reflection and landscape interaction induced by the poetry task for these 16 participants might have been associated with positive physiological relaxation effects. These findings demonstrated that the WP experimental design not only enhanced participants' perception of the physical features of natural landscapes but also integrated emotional responses to evoke subjectively emotional comfort. Moreover, the observed correlation between behavioral and physiological responses suggested a potential mechanistic link, though further research was needed to explore this relationship given the limitations of the current study.

These findings above align with the findings of previous research on meditation, namely that intentional attention control during nature-based mediation enhances awareness and strengthens connection with nature (Nisbet et al., 2019). Furthermore, our study suggested the potential to extend this understanding by indicating that structured proactive interventions in participants‘ viewing processes could help facilitate cognitive-affective outcomes within shorter timeframes. Some perspectives suggest that literary interventions in meditation practice may alter individuals' thought processes (Moffett, 1983). The results of the WP experiment proved this argument. However, other perspectives argue that Chinese Buddhism prescribes a vigilant attitude, pragmatically implemented through maintaining a certain degree of active control and sitting posture with eyes open, which may counteract the goals of meditation (Austin, 1999). Nevertheless, the findings of the present study suggested that such active control, when manifested through the practice of composing poetry integrated with visual experiences in the Chinese Buddhist tradition, could potentially yield positive outcomes (e.g., enhanced nature awareness and improved relaxation), which might serve as essential prerequisites for deepening meditative practice (Zhang, 2006). Therefore, the effects depend on the form of stimulus for active control, and how this stimulus can be used to better inspire further positive effects is a technical task worthy of further investigation.

## 5 Strengths and limitations

This study employed an empirical approach to examine the effectiveness of nature observation and literary creation in traditional Chinese Buddhist meditation practices. It used quantitative measurements to visualize and digitize the effects of these practices, providing evidence-based results for researchers interested in understanding or investigating Buddhist meditation traditions, as well as methodological references.

This study also has the following limitations. First, the research was conducted with a relatively small sample size consisting exclusively of Chinese participants familiar with Han Buddhist traditions. Given the considerable diversity of Buddhist practices worldwide (including Theravada, Tibetan, and Zen traditions), future investigations should employ more diverse participant populations to properly assess the cross-cultural generalizability of these findings. Second, the absence of a traditional control group (e.g., urban or indoor setting) limited our ability to rule out expectancy effects or placebo responses. Future studies should include active control conditions to isolate the unique contributions of landscape and poetry tasks. Additionally, the age and occupational differences between Experiments 1 and 2 were not statistically controlled and therefore a potential source of bias. For instance, older participants in Experiment 2 might have had prior exposure to poetry composition, potentially inflating the observed effects. These factors should be explicitly balanced in future replications. Third, as this study was a preliminary exploration, the methodology was limited to using a fixed poetic form to measure the effects of literary creation, rather than incorporating more diverse formats for a broader investigation. Consequently, the fixed five-line poetry format employed in this research may have influenced participants‘ initial impressions to some extent. As a result, this study could not conclusively determine whether poetic structure had a decisive effect on participants' psychological or physiological responses. Further research is needed to explore this question in greater depth. Finally, the experimental setting was confined to specific temple landscapes; follow-up studies could extend to natural environments in other Chinese Buddhist temples to assess broader applicability.

## 6 Conclusion

This study employed an evidence-based approach to conduct experiments, exploring the effects of the meditation traditions of Chinese Buddhist monks under the influence of indigenous Chinese philosophies, Taoism and Confucianism. It demonstrates that meditation is not merely a static inner practice but can also involve further interactions (e.g., engaging with nature, composing poetry) to achieve deeper mind-body harmony.

The results indicate that the incorporation of natural environments into meditation practices under Taoist influence has introduced a distinct visual experience. The water landscape was found to be especially effective in promoting deep engagement and relaxation owing to its scenic quality and visual appeal, potentially helping meditators reach a deeper state of relaxation more quickly, while close-up observation of rocks may better suit those who prefer detailed focus.

Furthermore, incorporating the Confucian-influenced literary meditation practice appeared to not only enhance landscape analysis but also potentially extend benefits beyond relaxation, possibly through facilitating cognitive activation via deeper thinking. The findings suggested that literary creation might function as both an attentional guidance tool and a cognitive enhancement strategy, which could contribute to deepening human-nature interaction. However, due to methodological constraints, Experiment 2 did not administer the POMS scale, precluding direct comparison of mood states between WP and NP conditions. While qualitative analysis of poetic content (e.g., positive emotional expression and reflection depth) and physiological data suggested potential psychological benefits, the absence of standardized mood measures required future studies to incorporate parallel psychological assessments across conditions to more rigorously interpret these effects and validate these exploratory findings. Therefore, we recommend that when task constraints preclude full-scale administration, future studies could employ concurrent mood measures (e.g., PANAS). This approach may effectively mitigate interference from irrelevant factors on the experimental measurement objectives (as observed in the WP experiment of this study).

This study provides preliminary empirical evidence for the scientific validity of traditional Chinese Buddhist meditation integrating landscape observation with literary creation, highlighting how individual preferences (e.g., expansive views vs. detailed focus) and task diversity (e.g., poetry composition) can inform personalized practice. Additionally, this study offers the following practical implications: First, natural scenes with greater visual appeal and aesthetic quality can elicit more favorable psychophysiological responses in participants. This finding can serve as a reference for landscape architects designing meditation spaces in the future; Second, it proposes a novel approach for modern mental health interventions by incorporating structured poetry tasks to enhance the cognitive benefits of nature-based therapy.

## Data Availability

The original contributions presented in the study are included in the article/supplementary material, further inquiries can be directed to the corresponding author.
